# Sphingomonas Paucimobilis Infections in Children: 24 Case Reports

**DOI:** 10.4084/MJHID.2013.040

**Published:** 2013-06-05

**Authors:** Nuri Bayram, İlker Devrim, Hurşit Apa, Gamze Gülfidan, Hande Namal Türkyılmaz, İlker Günay

**Affiliations:** 1 Department of Pediatric Infectious Diseases, Dr. Behçet Uz Children’s Hospital, İzmir, Turkey; 2 Department of Microbiology, Dr. Behçet Uz Children’s Hospital, İzmir, Turkey; 3 Department of Pediatrics, Dr. Behçet Uz Children’s Hospital, İzmir, Turkey

## Abstract

*Sphingomonas paucimobilis* is a causative agent of infection in immunocompromised patients, and healthcare-associated infection. Although the infections associated with *S.paucimobilis* occurs rarely, it has been encountered with increasing frequency in clinical settings. In the current study we reported clinical features of the children with *S.paucimobilis* infection, and the antimicrobial susceptibilities of the isolated strains among the patients.

This study was conducted in Dr. Behçet Uz Children’s Hospital, Turkey, during the period of January 2005 and December 2012. The medical records of pediatric patients with positive cultures for *S.paucimobilis* were reviewed.

*Sphingomonas paucimobilis* isolates were recovered from 24 pediatric patients. The median age was 4 years (ranging from 3 days infant to 15 years) and 58,3% were male. Eight (33,3%) of the patients were under 1 months of age. Among the patients; 13 (54,2%) infections were community related however 11(45.8%) infections were nosocomial infection. The median duration of hospital stay was 7 days (ranging from 4 to 22 days). The most effective antibiotics were fluoroquinolones, carbapenems, and trimethoprim/sulfamethoxazole.

This is the first largest study in children to evaluate the clinical features of *S. paucimobilis* infections. *Sphingomonas paucimobilis* may cause infections in both previously healthy and immunocompromised children. Although variable antimicrobial regimens were achieved to the patients, there was no attributable fatality due to *S.paucimobilis* infections due to the low virulence of the bacteria.

## Introduction

*Sphingomonas paucimobilis* is a Gram-negative bacillus that is emerging as an opportunistic pathogen.^1^ It is widely found in nature, especially in water and soil, and has been isolated from hospital environments such as distilled water, nebulizers, and multiple equipments used in medical care.^2^

It has been implicated as a causative agent of infections in immunocompromised patients, plus healthcare-associated infections. Although the infections associated with *S.paucimobilis* were reported to occur rarely, it has been more frequently reported in clinical settings. There are few published articles about the *S.paucimobilis* infections and majority of them are limited to case reports or reports with low number of cases.^3^–^8^ There is no published serial data about the infections related to *S.paucimobilis* in pediatric settings. Thus, in the current study we aimed to note the clinical features of the children with *S.paucimobilis* infections, and the antimicrobial susceptibilities of the isolated strains among the pediatric patients hospitalized in Dr. Behçet Uz Children’s Hospital, Turkey, during 2005 – 20012.

## Materials and Methods

### Data collection

This study was conducted in Dr. Behçet Uz Children’s Hospital, during the period of January 2005 and December 2012. Doctor Behçet Uz Children’s Hospital, Izmir, Turkey, is a 400-bed pediatric teaching tertiary hospital with 24-bed ICU and approximately 20 000 hospitalization per year.

The medical records of pediatric patients with positive cultures for *S.paucimobilis* were reviewed. The patients evidenced with infections were included in this study. Demographic and clinical data including age, gender, underlying diseases, presence of central venous catheter (CVC), use of mechanical ventilation, use of total parenteral nutrition (TPN), duration of hospitalization, antimicrobial chemotherapy, and clinical outcomes were collected.

#### Case definition

Hospital-associated infection was defined as isolation of *S.paucimobilis* obtained from sterile specimens that was not on incubation or present at the time of hospital admission, and that develops ≥48 hours after hospital admission or ≤48 hours after hospital discharge in conjunction with the presence of clinical symptoms or signs of infection without clinical evidence of bacteremia on admission. Community-acquired infection was defined as infections that occur in patients who have not been hospitalized recently or had a recent medical procedure.^9^

The source of bacteremia was decided based on clinical findings and bacterial culture results. Bacteremia without concomitant infectious focus was considered as primary bacteremia. Catheter-related bloodstream infection was defined as positive simultaneous blood cultures from the central venous catheter and peripheral vein yielding the same organism in the presence of at least one of the following; 1) Simultaneous quantitative blood cultures in which the number of CFU/mL isolated from blood drawn through the central catheter is ≥5-fold the number isolated from blood drawn peripherally 2) Positive semiquantitative (≥15 CFU/catheter segment) or quantitative (≥100 CFU/catheter segment) catheter tip cultures 3) Simultaneous blood cultures of equal volume in which the central blood culture has growth in an automated system ≥2 hours earlier than the peripheral blood culture.^10^

We defined initially inappropriate antibiotic therapy as occurring when the patient either was not administered an antibiotic within 48 hrs of infection or was treated with an antibiotic to which the isolated pathogen was resistant in vitro.

### Microbiology on culture samples

Bottles from each blood culture were placed in the BacT/ALERT (Biomerieux France) instrument and incubated for 7 days or until they signaled positive. A sample of blood from positive blood cultures was inoculated onto chocolate, EMB and blood agar plates and incubated at 37°C, 5% CO_2_, for up to 48 hours. *Sphingomonas paucimobilis* isolates were characterized as gram-negative rods that are yellow-pigmented, glucose nonfermenting, and weakly oxidase positive. The *S.paucimobilis* isolates were identified according to biochemical profiles established with the use of Vitek 2 compact system (bioMerieux, France). Antibiotic susceptibility test was also performed with Vitek 2 (bioMerieux, France) system for each isolate according to the manufacturer’s instructions and the Clinical and Laboratory Standards Institute’s (CLSI) criteria.

Isolates were considered as resistant if the MIC values were found to be for cefuroxime ≥64 μg/mL, for 3rd generation cephalosporins ≥64 μg/mL, for ampicillin ≥128 μg/mL, for piperacillin ≥128 μg/mL, for amikacin ≥64 μg/mL, for ciprofloxacin ≥4 μg/mL, for trimethoprim/sulfamethoxazole ≥4/76 μg/mL, and for imipenem ≥16 μg/mL. If the MIC values were detected for cefuroxime ≤8 μg/mL, for 3rd generation cephalosporins ≤8 μg/mL, for ampicillin ≤16 μg/mL, for piperacillin ≤16 μg/mL, for amikacin ≤16 μg/mL, for ciprofloxacin ≤1 μg/mL, for trimethoprim/sulfamethoxazole ≤2/38 μg/mL, and for imipenem ≤4 μg/mL, the strains were accepted as susceptible.

## Results

*Sphingomonas paucimobilis* isolates were recovered from 33 pediatric patients during the study period. Among these, 9 patients were excluded from the study because of not compatible with definition of infection. As a result, a total of 24 clinically compatible patients were evaluated in the study.

The median age was 4 years (ranging from 3 days infant to 15 years) and 14 (58.3%) of the patients were male. Eight (33,3%) of the patients were ≤1 month of age. Eleven of 24 patients had underlying diseases and co-morbidities including 4 acute lymphocytic leukemia with neutropenia, 2 surgical co-morbidity (duodenal atresia and imperforated anus), 1 Down syndrome, 1 steroid induced immunosuppression due to post streptococcal acute glomerulonephritis, 1 burn injury, and 2 history of prematurity. Clinical syndromes included 20 patients with bacteremia and intravascular catheter related bloodstream infections in 2 patients; one central nervous system infection, and 1 urinary tract infection. Among 24 patients; 13 (54,2%) infections were community related, however 11 (45.8%) infections were nosocomial infection. Demographic and clinical characteristics and procedures applied on the patients enrolled in the study were reviewed in Table 1. The median duration of hospital stay was 7 days ranging from 4 to 22 days.

**Figure 1:**
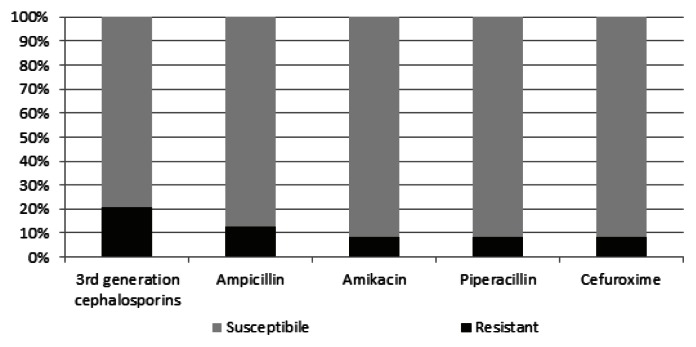
The antimicrobial susceptibility patterns of *Sphingomonas paucimobilis* isolated from clinical specimens.

The antimicrobial resistance patterns of the isolates of *S.paucimobilis* were reviewed in Figure 1. Among the patients, 9/24 (37,5%) of the isolates were resistant to at least one of the antibiotics. Four of the nine-resistant bacteria were found in newborns. The most resistant pattern identified against to 3rd generation cephalosporin (5/24, 20,9%). Resistance to other antibiotics were as following; ampicillin resistance in 3 patients (12,5%), amikacin resistance in 2 patients (8,3%), piperacillin resistance in 2 patients (8,3%), and cefuroxime resistance in 2 patients (8,3%). All isolates were susceptible to imipenem, ciprofloxacin, and trimethoprim/sulfamethoxazole.

Although initially inappropriate antibiotic therapy were revealed in five patients, there was no attributable fatality due to *S.paucimobilis* infections and in all the patients negative cultures were achieved with variable antimicrobial regimens.

## Discussion

*Sphingomonas paucimobilis* isolates have been recovered from multiple sources of hospital environments. Although some case reports or case series of *S. paucimobilis* infection have been published for adult patients, the clinical features of *S. paucimobilis* infections are still less well known especially in pediatric settings. Thus, we retrospectively evaluated the clinical features and treatment options associated with infections caused by *S. paucimobilis* in children.

Most of the *S.paucimobilis* infections reported in the literature have been health-care associated infections.^11^–^13^ But contrary to the recent publications, in this study, most of the infections were revealed as community-acquired infections. Thirteen patients (54,2%) infected with *S. paucimobilis* were previously healthy children and neither had any healthcare-associated risk factors or underlying diseases. Due to widespread distribution of the *S.paucimobilis* in both natural environment and hospital settings, this result was not surprising for us. Similarly, some community-acquired infections attributed to *S.paucimobilis* were also reported.^12^,^14^ But, clinical importance of the community-acquired infections has not been not well-understood yet due to the limited number of cases.

Furthermore, neonatal patients may be more susceptible to *S.paucimobilis* infections. In the current study, 8 patients (33,3%) were under 1 month age. In the literature, only two reports had denoted *S.paucimobilis* infection in newborns. Lemaitre et al. reported tracheal colonization with *S.paucimobilis* in mechanically ventilated neonates due to contaminated ventilator temperature probes, and Mutlu et al. reported an outbreak of *S.paucimobilis* septicemia in a neonatal intensive care unit.^15^,^16^

The *S.paucimobilis* isolates in this study exhibited antibiotic susceptibility trends that were different from those in other studies. Previous reports suggested that third generation cephalosporins or aminoglycosides were best choice of treatment of *S.paucimobilis* infections.^2^,^11^,^12^ However, 20.0% of the isolates in our study were resistant to cefotaxime, and 13.6% were resistant to amikacin. Carbapenems were the most effective therapy in our study. These differing results reinforce the need to treat these infections with individualized antibiotic therapy, guided by the in vitro susceptibility of each clinical isolate.

All patients received initial antibiotic therapies; ampicillin or ampicillin/sulbactam in 5 patients, second generation cephalosporins in 4 patients, 3rd generation cephalosporins in 5 patients, aminoglycosides in 4 patients (in combination with β–lactam agent), piperacillin/tazobactam in 5 patients, meropenem in 5 patients, and vancomycin in 3 patients. Inappropriate initial antibiotic therapy were detected in 5 patients (20,8%). However, outcome of all patients were favorable despite inappropriate initial therapies. This circumstance may be related with the low virulence of *S.paucimobilis* that was emphasized in previous studies.^11^,^12^,^14^ For the patients diagnosed as CR-BSI, the central catheters were not required to remove for treatment. No cases of death have been recorded in the literature related to S. paucimobilis.

There are no standardized and recommended for therapies of *S.paucimobilis* infections. Thus, antibiotic treatment rested on clinical experience. The duration of the treatment was 7 – 13 days according to the clinical response of the patients.

Despite the fact that we detected only 24 patients clinically compatible with *S. paucimobilis* infection, there are only five reports in literature about *S. paucimobilis* infections including more than 10 patients.^11^,^12^,^14^,^16^ Moreover, only one of these reports was in pediatric settings with outbreak of *S.paucimobilis* septicemia in a neonatal intensive care unit. Thus, to our knowledge, this is the first largest study in children to evaluate the clinical features of *S. paucimobilis* infections.

In conclusion, *S. paucimobilis* may cause infections in both previously healthy and immunocompromised children. Beside the infections associated with *S.paucimobilis* occurs rarely in clinical settings; it has being increasingly reported over years. Due to its low virulent features of bacteria, it is not associated with serious life-threatening infections; however these bacteria will increase its role in health-care settings.

## Figures and Tables

**Table 1:** Demographic and clinical characteristics of the patients enrolled in the study.

Cases	Age	Gender	Underlying conditions	Acquired source	Infectious foci	Empirical antibiotic therapies	Applied procedures (duration)
**1**	4 months	Female	None	Community	UTI	Cefuroksime	-
**2**	1 years	Male	imperforate anus	Hospital	Primary bacteremia	Ceftazidime	TPN (3 days)
**3**	23 days	Male	duodenal atresia	Hospital	Primary bacteremia	Cefotaxime, Amikacin	TPN (8 days)
**4**	3 years	Male	None	Community	Primary bacteremia	Cefotaxime	-
**5**	10 years	Female	ALL, neutropenia	Hospital	CR-BSI	Piperacillin/tazobactam	CVC (30 days)
**6**	12 years	Male	PSAGN	Community	Primary bacteremia	Cefuroxime	-
**7**	4 years	Male	Burn injury	Hospital	CR-BSI	Meropenem, Amikacin	MV (10 days), CVC (22 days)
**8**	8 years	Female	ALL, neutropenia	Hospital	Primary bacteremia	Piperacillin/tazobactam	-
**9**	18 days	Male	prematurity	Community	Primary bacteremia	Ampicillin, Amikacin	-
**10**	8 days	Female	prematurity	Hospital	Primary bacteremia	Meropenem, Vancomycin	TPN (8 days)
**11**	7 years	Female	None	Community	Primary bacteremia	Cefuroxime	-
**12**	10 years	Male	None	Community	CNS	Cefotaxime, Vancomycin	-
**13**	9 years	Male	Down syndrome	Community	Primary bacteremia	Meropenem, Vancomycin	-
**14**	1 month	Male	None	Community	Primary bacteremia	Ampicillin/sulbactam	-
**15**	12 years	Female	None	Hospital	Primary bacteremia	Piperacillin/tazobactam	-
**16**	1 month	Female	None	Community	Primary bacteremia	Ampicillin/sulbactam	-
**17**	11 years	Male	None	Hospital	Primary bacteremia	Meropenem	-
**18**	4 years	Male	ALL, neutropenia	Hospital	Primary bacteremia	Piperacillin/tazobactam	CVC (96 days)
**19**	15 years	Female	None	Community	Primary bacteremia	Cefotaxime	-
**20**	1 years	Female	None	Community	Primary bacteremia	Cefuroxime	-
**21**	5 years	Male	ALL, neutropenia	Hospital	Primary bacteremia	Piperacillin/tazobactam	-
**22**	3 days	Male	None	Community	Primary bacteremia	Ampicillin, Amikacin	-
**23**	23 days	Male	None	Community	Primary bacteremia	Ampicillin/sulbactam	-
**24**	27 days	Female	None	Hospital	Primary bacteremia	Meropenem	-
